# Association study between genetic polymorphisms in MTHFR and stroke susceptibility in Egyptian population: a case–control study

**DOI:** 10.1038/s41598-023-50277-z

**Published:** 2024-01-02

**Authors:** Omali Y. El-khawaga, Mohammed F. Al-azzawy, Aliaa N. El-Dawa, Afaf M. ElSaid, Wessam Mustafa, Mariam Saad

**Affiliations:** 1 Biochemistry Division, Chemistry Department, Faculty of Science, Mansoura University, Mansoura, 35516 Egypt; 2 Genetic Unit, Department of Pediatrics, Faculty of Medicine, Mansoura University, Mansoura, 35516 Egypt; 3 Neurology Department, Mansoura University Hospital, Mansoura, 35516 Egypt

**Keywords:** Biochemistry, Genetics, Risk factors

## Abstract

Stroke is a major global disability cause, and genetic variables for multifactorial illnesses like stroke are crucial for precision medicine. The purpose of this study is to see if genetic variants in the MTHFR gene are associated with a higher risk of ischemic stroke among the Egyptian population. A case–control study was conducted at Mansoura University Hospital, involving 100 stroke patients and 150 healthy volunteers as the control group. Peripheral blood genomic DNA was isolated and single-nucleotide polymorphisms were genotyped using ARMS-PCR. The CT and TT genotypes of the C677T gene polymorphism exhibited substantial risks for having stroke disease [(OR 3.856; P ≤ 0.001); (OR 4.026; P ≤ 0.001), respectively]. The T allele was significantly more prevalent among patients compared to controls. (OR 2.517; (P = 0.001)). The over-dominant and dominant models demonstrated a substantial relationship between stroke groups at risk of developing stroke but not the Recessive model. An extensive connection was found between the MTHFR A1298C and stroke danger in three different inheritance models: dominant (CC + CA vs. AA), over-dominant (AA + CC vs AC), and allelic (C allele) (P < 0.001). A highly significant difference in blood pressure, total cholesterol, and triglycerides levels was found between patients and control. While there was no meaningful link discovered between genetic polymorphism with SBP, DBP, TG, LDL, VLDL among stroke group (P > 0.05 for each) except the CC genotype that was significantly associated with lower levels of TC and HDL when compared to CT + TT genotypes. The study evaluates a strong link among MTHFR mutations in genes and the probability to get stroke. The research significantly supports the use of MTHFR ((rs1801133) and (rs1801131) variations in stroke prediction.

## Introduction

Stroke is currently the second-greatest cause of adult death and the third-greatest cause of disability^1^. Based on population data conducted in EGYPT, stroke occurs at a higher incidence than in most Western countries and at a younger age, reaching 20.5%^2^. Knowing risk factors is necessary for understanding the etiology of stroke. Traditional hazards for ischemic strokes, including diabetes, hypertension, atrial fibrillation, and tobacco use, have been widely studied, although they account for just a modest portion of stroke risk. Considering that many stroke patients do not have those risk factors, many previously recognized causes of stroke do not adequately explain the physiology of stroke^3^. Stroke represents a complicated illness with a considerable genetic predisposition to ischemic stroke, in accordance with findings from twin and family aggregation stroke research^4^.

The methylenetetrahydrofolate reductase (MTHFR) is a folate-metabolizing enzyme that produces the 5-methyl THF required for the re-methylation of homocysteine to methionine, which is ultimately employed to produce the global methyl donor, SAM^5^. The MTHFR gene has been located on chromosome 1p36.3, which has a complicated genomic structure^5^. Mutations that occur in the MTHFR gene have been linked to an elevated probability of stroke^6^. C677T (rs1801133) and A1298C (rs1801131) polymorphisms are considered the two most important polymorphisms that may influence enzyme activity. The C677T variant is a point alteration that causes a cysteine to thymine nucleotide substitution at point 677 on the MTHFR gene, resulting in an alanine to valine substitution in the MTHFR enzyme, which is the most often researched genetic variation and has the strongest correlation with elevated hyper homo-cysteinemia (tHcy). This polymorphism, which is widespread in the general population but varies by ethnic origin, appears to be implicated in the genetic etiology of numerous disorders^7–9^. However, research on the connections between MTHFR genetic variations and the danger of stroke has yielded inconclusive results^10,11^. Except for C677T, a second prevalent mutation found in the gene, A1298C, leads to markedly lower catalytic activity of the enzyme, despite the fact that few research has focused on that polymorphism^12–14^. The aforementioned variants have been linked to the risk of chronic myeloid leukemia (CML)^7^, hepatocellular carcinoma^5^, and breast cancer^8^, as well as the pathogenesis of lipid disorders related to type 2 diabetes^9^. However, the role of these variations in the risk of ischemic stroke among Egyptians was not previously studied. Consequently, this study aims to evaluate whether MTHFR C677T and A1298C polymorphisms are associated with proven ischemic stroke patients through a case–control study in Egyptians, as well as their relationship with the biochemical parameters of the patients.

## Results

The current study is a case–control study with 150 control volunteers and 100 non-relevant stroke survivors. Patients varied in age from 19 to 88 years old, with a mean age of 61.9. They were made up of 60% men and 40% women. The formula developed by Hardy–Weinberg indicated that all genotypes investigated in the control and stroke groups were in HW equilibrium, since no significant discrepancies between observed and anticipated counts were identified in either group and there is no statistically significant difference in age (P = 0.839) or sex (P = 0.253) between the two groups. A comparison of blood pressure levels and laboratory findings was performed between patients and healthy volunteers. Stroke patients had substantially higher SBP (P = 0.017), cholesterol (P = 0.020), high-density lipoprotein (HDL) (P < 0.001), and low-density lipoprotein (LDL) (P < 0.001) than controls. The outcomes also demonstrate that stroke patients had significantly reduced DBP, triglyceride (TG), and very low-density lipoprotein (VLDL) levels (P < 0.001, for each) in comparison to the control healthy group (Table 1).

**Table 1:** Comparison of demographic data among stroke patients and control group.

	Control n = 150	Stroke cases n = 100	Test (p)	*P*
Age (years)	64 (19–88)	64 (19–88)	T = 0.204	0.839
SEX (male/female)	79/71	60/40	X^2^ = 1.307	0.253
SBP (mmHg)	124 (100–198)	130 (90–190)	T = 2.413	0.017
DBP (mmHg)	80 (58–89)	80 (50–100)	T = 3.740	< 0.001
TG (mg/dl)	173 (151.55–198)	119 (26–887)	U = 11,104.0	< 0.001
TC (mg/dl)	162.2 (100–198)	167.5 (72–302)	T = 2.359	0.020
HDL-C (mg/dl)	30.23 (23–41)	35.6 (25–60)	T = 8.493	< 0.001
LDL-C (mg/dl)	34.6 (30.31–39.6)	107.1 (30.3–222.4)	U = 471.5	< 0.001
VLDL-C (mg/dl)	34.6 (30.4–39.6)	23.8 (5.2–177.4)	U = 11,112.0	< 0.001

*P* probability, *T* student test, *X*^*2*^ chi square test, *U* Mann–Whitney.

### Distribution of MTHFR (rs1801133) and (rs1801131) genetic polymorphisms in controls compared to stroke patients

MTHFR A1298C and C677T gene variants were examined in 100 stroke patients and 150 healthy controls. Based on the calculated *P* values, there was no substantial deviation from Hardy–Weinberg equilibrium, implying that in the absence of any evolutionary factors, the allele and genotype frequencies of the indicated SNPs in the examined population are not predicted to change from generation to generation. Table 2 reveals significant findings for both variations among patients and controls.

**Table 2:** The association between mentioned SNPs and risk of stroke in allelic, co-dominant, dominant and recessive models.

	Model		Control n = 150	Stroke n = 100	P-value	OR (95% CI)
			n (%)	n (%)		
MTHFR C677T	General	CC	70 (46.7)	8 (8.0)		Reference
		CT	71 (47.3)	81 (81.0)	< 0.001	3.856 (2.518–5.905)
		TT	9 (6.0)	11 (11.0)	< 0.001	4.026 (2.066–5.905)
	Recessive	CC + CT Vs TT	141 (94)	89 (89)		Reference
			9 (6.0)	11 (11.0)	0.159	1.511 (0.850–2.685)
	Over-dominant	CC + TT Vs CT	79 (52.7)	19 (19)		Reference
			71 (47.3)	81 (81.0)	< 0.001	2.576 (1.819–3.647)
	Dominant	CC Vs CT + TT	70 (46.7)	8 (8.0)		Reference
			80 (53.3)	92 (92)	< 0.001	3.875 (2.544–5.803)
	Allelic	C-Allele	211 (70.3)	97 (48.5)		Reference
		T-Allele	89 (29.7)	103 (51.5)	< 0.001	2.517 (1.736–3.651)
MTHFR A1298C	General	AA	68 (45.3)	11 (11.0)		Reference
		AC	73 (48.7)	88 (88.0)	< 0.001	3.323 (2.238–4.933)
		CC	9 (6.0)	1 (1.0)	0.728	0.821 (0.269–2.500)
	Recessive	AA + AC Vs CC	141 (94)	99 (99)		Reference
			9 (6.0)	1 (1.0)	0.083	0.346 (0.119–1.011)
	Over-dominant	AA + CC Vs AC	77 (51.3)	12 (12)		Reference
			73 (48.7)	88 (88.0)	< 0.001	3.390 (2.318–4.959)
	Dominant	AA Vs AC + CC	68 (45.3)	11 (11.0)		Reference
			82 (54.7)	89 (89)	< 0.001	3.111 (2.102–4.606)
	Allelic	A-Allele	209 (69.7)	110 (55.0)		Reference
		C-Allele	91 (30.3)	90 (45.0)	< 0.001	1.879 (1.296–2.725)

*OR* odd ratio, *P* probability; *P* < 0.05 significant, Regression analysis was used.

**Figure 1:**
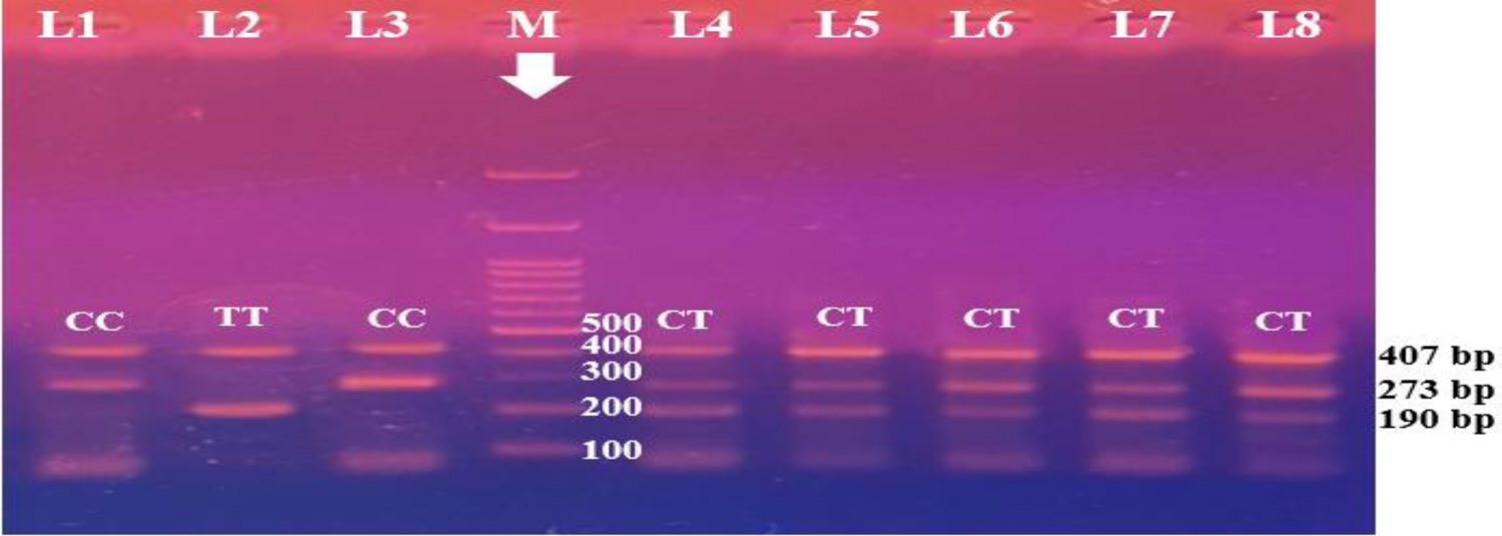
Tetra-ARMS PCR electrophoretic pattern of the MTHFR C677T product, where each lane represents one participant. M stands for DNA marker (100 bp). The internal control is shown by the 407 bp band. Based on the primer, specific 273 bp bands represent the wild (C) allele, and specific 193 bp bands represent the mutant (T) allele. CT heterozygous is represented by lanes 4, 5, 6, 7 and 8. Lanes 1 and 3 indicate wild homozygous (CC), where the T allele is absent from the lane and the C allele is present at 273 bp. Lane 2 and represents mutant homozygosity (TT), with the T allele appearing at 193 bp and the C allele absent.

**Figure 2:**
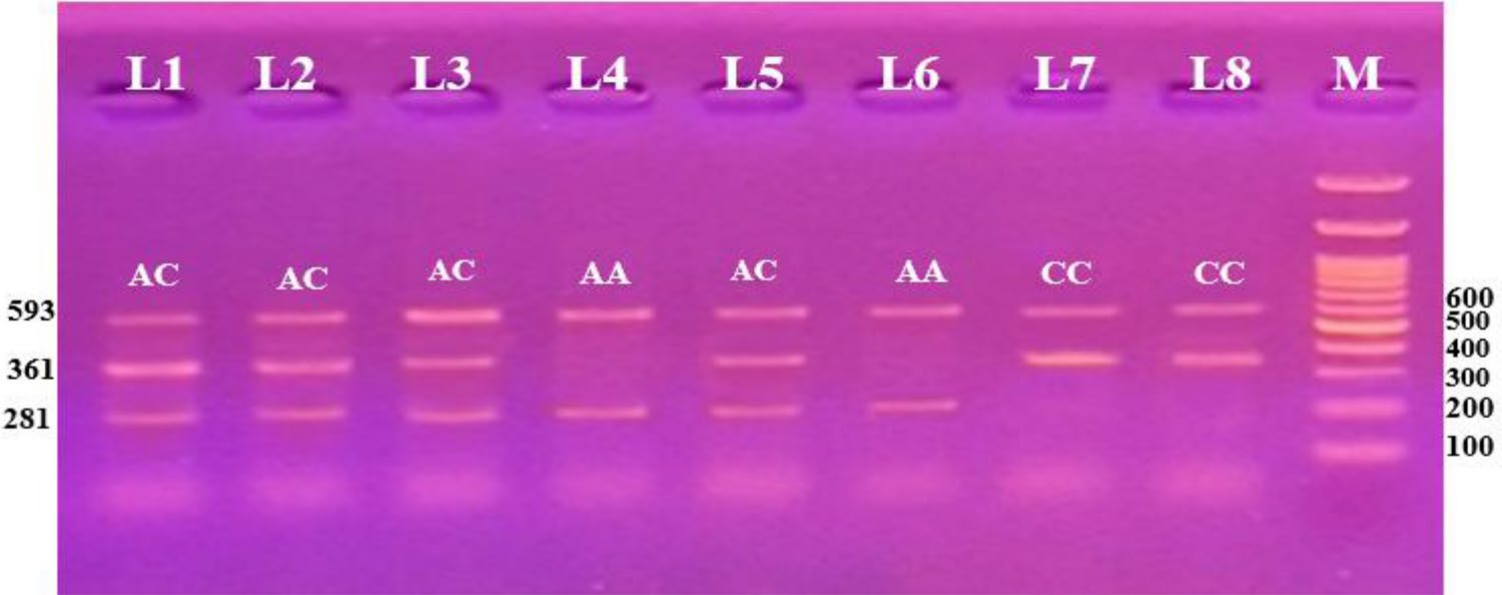
Tetra-ARMS PCR electrophoretic pattern of the MTHFR A1298C product, where each one lane represents one participant. M stands for DNA marker (100 bp). The internal control is shown by the 593 bp band. Based on the primer, specific 281 bp bands represent the wild (A) allele, and specific 361 bp bands represent the mutant (C) allele. AC heterozygous is represented by lanes 1, 2, 3 and 5. Lanes 4 and 6 indicate wild (AA) homozygous, where the C allele is absent from the lane and the A allele is present at 281 bp. Lanes 7 and 8 and represent mutant (CC) homozygosity, with the C allele appearing at 361 bp and the A allele absent.

For C677T SNP, the CC homozygous genotype was seen in 8% of patients and 46.7% of healthy controls, CT heterozygous genotype was greater in patients (81% vs. 47.3%), and TT homozygous genotype was higher in patients (11% vs. 6%). When compared to the control group, the CT and TT genotypes of the MTHFR C677T gene polymorphism exhibited substantial risks for having stroke disease [(OR 3.856; P ≤ 0.001); (OR 4.026; P ≤ 0.001), respectively]. Furthermore, the over-dominant model (OR 2.576; P ≤ 0.001) and dominant model (OR 3.875; P ≤ 0.001) demonstrated a considerably greater frequency among stroke groups at risk of developing stroke. When recessive inheritance was assumed, the frequencies of TT vs TC + CC were insignificant among patients and controls. The T allele was significantly more prevalent among patients compared with controls ([OR] 2.517; (P = 0.001)) (Fig. 1). For the A1298C, evaluations revealed that the AA homozygous genotype was lower in patients (11%) than in the control group (45.3%). The AC heterozygous genotype was found in 88% of stroke patients compared to 47% of controls, indicating a link with stroke patients. The CC homozygous genotypes have a lower incidence of stroke (1%) than the control group (6%). We also found a strong association between this SNP and stroke risk in three different inheritance models: dominant (CC + CA vs. AA), over-dominant (AA + CC vs AC), and allelic (C allele) (*P* < 0.001 for each), while recessive (CC vs CA + AA) showed no significant differences between cases and controls (*P* > 0.05) (Fig. 2).

### Association of MTHFR genotype polymorphisms with other parameters

The risk factor profile revealed that hypertension was the most common risk factors in patients found in 70% Followed by and smoking in 46% of cases. The relationship between demographic, clinical, and laboratory factors and MTHFR C677T polymorphisms in stroke patients is shown in Table 3. No significant association was found regarding MTHFR with gender (*P* = 0.690) or age (*P* = 0.690). Among all studied stroke patients. Laboratory parameters investigated showed that the CC genotype was significantly associated with lower levels of TC (*P* = 0.046) and HDL (*P* = 0.028) when compared to CT + TT dominant genotype. While no significant association was found between genetic polymorphisms and SBP, DBP, TG, LDL, or VLDL regarding stroke groups (*P* > 0.05 for each) as well as no correlation with smoking status of stroke patients.

**Table 3:** The association between the demographic, clinical and laboratory variables and the MTHFR C677T polymorphisms in stroke patients.

	CT n = 81	CT + TT n = 92	CC n = 8	TT n = 11	Test (p)	*P*_*1*_	Test (p)	*P*_*2*_
SBP (mmHg)	130 (100–190)	130 (90–190)	135 (120–160)	140 (90–160)	F = 0.553	0.577	T = 0.960	0.339
DBP (mmHg)	80 (60–100)	80 (50–100)	80 (70–90)	80 (50–100)	F = 0.739	0.48	T = 0.101	0.921
TG (mg/dl)	120 (26–887)	121.5 (26–887)	105.5 (62–231)	131 (98–276)	H = 0.632	0.729	U = 400.0	0.684
TC (mg/dl)	16 7 (80–302)	169.5 (80–302)	126 (72–196)	190 (126–253)	F = 2.821	0.044	T = 2.017	0.046
HDL-C (mg/dl)	30.9 (28.6–37.8*)*	36 (25–60)	30.9 (28.6–37.8)	36 (28–50)	F = 2.740	0.046	T = 2.225	0.028
LDL-C (mg/dl)	105.6 (30.3–222.4)	108.9 12.4–222.4)	70.6 (31–146)	128.9 (45.2–165.8)	H = 4.131	0.127	U = 507.5	0.076
VLDL-C (mg/dl)	105.6 (30.3–222.4)	24.3 (5.2–177.4)	21.1 (12.4–46.2)	128.9 (45.2–165.8)	H = 0.632	0.729	U = 400.0	0.684
Gender								
Male	52(64.2%)	58 (63%)	2 (25%)	6 (54.5%)	X2 = 4.814	0.092	X2 = 4.438	0.057
Female	29 (35.8%)	34 (37%)	6 (75%)	5 (45.5%)				
Smoking status								
Absent	6(75%)	41 (50.6%)	7 (63.6%)	48 (52.2%)	2.205	0.332	1.544	0.214
Present	2 (25%)	40 (49.4%)	4 (36.4%)	44 (47.8%)				

*OR* odds ratio, *CI* confidence interval, *P*_*1*_ comparison between CT, CC and TT, *P*_*2*_ comparison between CT + TT versus CC, *F* ANOVA, *T* student t test, *U* Mann Whitney test, *X2* chi square test.

Table 4 depicts the correlation of MTHFR A1298C genetic variation with different variables in all stroke patients. There was no significant relationship detected between MTHFR A1298C genotypes and patients regarding age or gender. Also, there was no significant link identified between MTHFR A1298C variation and SBP, DBP, TG, TC, HDL, LDL, VLDL or smoking status among stroke groups (P > 0.05 for each).

**Table 4:** The association between the demographic, clinical and laboratory variables and the MTHFR A1298C polymorphisms in stroke patients.

	AC + CC n = 89	AA n = 11	Test (*P)*	*P*
SBP (mmHg)	130.0 (100.0–190.0)	130.0 (90.0–160.0)	T = 0.302	0.764
DBP (mmHg)	80.0 (50.0–100.0)	80.0 (60.0–100.0)	T = 0.594	0.554
Triglyceride (mg/dl)	118.0 (26.0–887.0)	131.0 (62.0–578.0)	U = 474.5	0.869
Total cholesterol (mg/dl)	173.0 (80.0–302.0)	145.0 (72.0–284.0)	T = 1.290	0.2
HDL-C (mg/dl)	35.6 (25.0–60.0)	35.4 (28.0–39.0)	T = 1.078	0.284
LDL-C (mg/dl)	110.8 (12.40–222.40)	90.2 (31.0–133.0)	U = 638.5	0.101
VLDL-C (mg/dl)	23.6 (5.20–177.40)	26.2 (12.40–115.60)	U = 474.5	0.869
Gender				
Male	52 (58.4%)	8 (72.7%)	X^2^ = 0.834	0.518
Female	37 (41.6%)	3 (27.3%)		
Smoking status				
Absent	49 (55.1%)	5 (45.5%)	X2 = 0.363	0.547
Present	40 (44.9%)	6 (54.5%)		

*OR* odds ratio, *CI* confidence interval; *P* < 0.05 significant, *T* student t test, *U* Mann Whitney test, *X2* chi square test.

### Association of MTHFR C677T-A1298C haplotypes among studied groups

MTHFR C677T-A1298C haplotypes were calculated; the CA genotype showed the highest prevalence, while the CC genotype showed the lowest prevalence among stroke groups. In the healthy group, the CA genotype showed the highest prevalence, while the TC genotype showed the lowest prevalence. As depicted in Table 5, TA and TC haplotypes showed significantly higher prevalence among cases in comparison to the control group (P = 0.022 and < 0.001, respectively), with a higher risk of developing stroke (OR 1.622 and 2.656, respectively).

**Table 5:** Comparison of MTHFR C677T- A1298C haplotypes’ frequencies between stoke patients and controls.

MTHFR haplotypes C677T-A1298C	Stroke	Control	P	OR	95% CI
CA	0.285	0.489	–	1	Reference
TA	0.265	0.208	0.022	1.622	1.073–2.452
CC	0.200	0.215	0.209	1.319	0.856–2.031
TC	0.250	0.089	< 0.001	2.656	1.635–4.314

*OR* odds ratio, *CI* confidence interval; *P* < 0.05 significant; Regression analysis was used.

## Discussion

Mutations in the MTHFR gene, which is involved in homocysteine metabolism, cause increased homocysteine (hcy) buildup in the circulation. As a result, a change in the function of the MTHFR pathway increases the risk of cerebrovascular illness^15–17^. To develop precision healthcare, genetic factors for complex and polygenic disorders like stroke must be identified and verified. This gene’s genomic variations have been linked to a variety of human illnesses, including malignancies^18^, type 2 diabetes^19^, and hypertension^20^.

The MTHFR gene is located on p36.3 chromosome 1 in humans. Over 40-point mutations in the MTHFR gene have been found to date, with C677T (rs1801133) and A1298C (rs1801131) appearing to have the most clinical importance linked to an increased risk of ischemic stroke in certain populations^21^. The current research used a PCR-ARMS approach to investigate the relationship between both SNPs in the MTHFR gene (A1298C and C677T) and the possibility of stroke.

The study results indicated that the rs1801133 and rs1801131 SNPs were linked to a higher risk of stroke for the Egyptian population. MTHFR (rs1801133), which involves a C-to-T exchange at position 677, leads to alanine-to-valine conversion. We observed that stroke cases had a higher incidence of TC, TT genotype, and T allele (*P* < 0.05 for each) and an increased risk of getting stroke (OR > 1 for each). This finding is consistent with a prior study that indicated that missense mutations reduce normal MTHFR enzyme activity by roughly 70% and 35% in TT and CT genotype carriers, respectively^22^. A single marker study for the MTHFR 677 C>T polymorphism revealed a genotypic and allelic correlation with stroke^10^. Previous epidemiological research has found that mutations in the SNP C677T location are related to an increased risk of strokes^23^. In certain groups, the T allele of this SNP is also correlated with raised homocysteine levels and an elevated risk of stroke^21^. A meta-analysis found that the TT genotype of C677T had a 1.84-fold higher risk of hemorrhagic stroke than the CC genotype, and subgroup studies by ethnicity confirmed that this connection occurred in both Asian as well as Caucasian populations^24^. The results are consistent with the current study in the importance of the mutation with the increased risk factor in our research due to the difference in ethnicities. Although numerous studies focused on the relationship between the MTHFR C677T variant and stroke danger, the results differed, which might be attributed to differences in demographic groupings and sample size. Another meta-analysis of 152,797 people revealed no link between SNP 677 C>T and ischemic stroke^25^. According to research conducted in the European population, rs1801133 is associated with white matter hyperintensity volume and lacunar stroke, but not with the other stroke subtypes^26^. A meta-analysis also discovered no relationship between the rs1801133 C>T genetic variant and the probability of carotid dissection, an acknowledged cause of stroke^27^. Consequently, research into the relationship between these polymorphisms and stroke has been unclear.

Concerning the (rs1801131) variation, a frequent mutation in the MTHFR gene results in the conversion of adenine to cytosine and also a decrease in MTHFR enzyme activity. The AC genotype and C allele have a highly significant (*P* < 0.001) connection with stroke patients, with an odd ratio larger than 1 as well as the dominant (CC + CA vs. AA) and the over-dominant (AA + CC vs. AC) models. Numerous studies have been undertaken to determine the effect of the MTHFR A1298C mutation on the probability of ischemic stroke; nevertheless, the findings have been conflicting^25,28^. In an Indian group, Kumar et al. discovered that the CC genotype was significantly associated with stroke-related conditions compared to the AA genotype, but not in a Scottish sample^21^. It is beneficial to execute haplotype analysis, which is more accurate for genotype–phenotype correlations than single SNPs. Also, analyzing the biomarkers in groups is more useful than assessing them individually. Furthermore, haplotype analysis may be more revealing than studies of individual markers separately, especially when many markers on a single chromosome are used to determine their connection with a disease^29^. MTHFR C677T-A1298C haplotypes were calculated. We revealed that the frequencies of the CC genotype at the SNPs rs1801131 site and rs1801133 site in the MTHFR gene were less frequent in stroke cases, likely signifying that they were protective variants in the disease condition of stroke. Moreover, TA and TC haplotypes were significantly associated with the risk of developing an ischemic stroke.

Modifiable risk factors like smoking, hypertension, and hyperlipidemia are critical since interventions focused on lowering these variables can lessen the incidence of stroke. The examination of emerging risk factors is still an active topic of research^3^. Hypertension is a big public health concern. Many writers have examined hereditary genetic changes in the MTHFR gene with regard to the dangers of hypertension, with inconsistent results^6^. We found no evidence of a link between MTHFR A1298C or C677T genetic variants and hypertension. Many researchers have stated that the C677T polymorphism has been acknowledged as a risk indicator for hypertension in many ethnic groups^30^. Moroccan research discovered that (677TT) carriers had a higher risk of hypertension (OR 5.4, *P* = 0.008)^6^. Yan et al. also found a substantial connection between MTHFR C>T and the possibility of hypertension in Asians, Caucasians, and Chinese participants in a meta-analysis^31^. The 5–10-mTHFR gene is linked to 40–60% of plasma lipid phenotypic variation, perhaps contributing to dyslipidemia susceptibility, which is a symptom of stroke illness^32^. Previous research has connected MTHFR polymorphisms with hypertriglyceridemia, higher levels of total cholesterol, and LDL-c for the C677T variant^32,33^. There was a substantial association with decreased levels of HDL as well as TC relative to the CT + TT genotypes in the C677T variation in stroke patients. Gay et al. found similar results in the Chinese^34^ and Moroccan populations^6^. Szolnoki et al. discovered a synergistic impact between the MTHFR 677TT genotype and smoking in ischemic stroke patients^35^. However, because of the small size of the studied cohort and the ethnic differences, we were unable to find any evident association between the examined mutations and smoking patients.

Finally, this is the first case–control study to investigate whether MTHFR is distributed differently in stork- and healthy Egyptians. MTHFR polymorphisms (rs1801131) and (rs1801133) were found to be significantly associated with individual susceptibility to IS, supporting the use of these MTHFR variations in predicting stroke, which may aid in early detection and lead to an increased chance of survival among the Egyptian people. The research has limitations due to its limited sample size, the absence of homocysteine testing, and the complexity of stroke. It should be viewed with caution, and bigger, larger-scale research might give greater insight into the involvement of potential genes in stroke risk.

## Methods

### Participants

The comparison research encompassed 100 adult patients, whose ages ranged from 19 to 88 suffered from ischemic strokes at Mansoura University Hospital (MUH) from June 2022 to April 2023 and 150 age and sex-matched healthy people. Cardiologists diagnosed hypertension in patients based on the guidelines established by the International Society of Hypertension (systolic blood pressure (SBP) ≥ 140 mmHg and diastolic blood pressure (DBP) ≥ 90 mmHg)^36^. The occurrence of ischemic stroke in individuals was established using brain CT or MRI. The Questionnaire for Verifying Stroke-Free Status (QVSFS) was used to determine stroke-free status in people who had normotensive blood pressure and no family history of cardiovascular disease or diabetes. The study excluded patients with transient ischemic stroke, cerebral embolism, hemorrhagic infraction, cerebral hemorrhage, subarachnoid hemorrhage, arteritis, tumors, aneurysms, concurrent liver and renal illness, thyroid disease, or rheumatologic disease. Controls who were on any type of medicine, had surgery, or had experienced trauma in the previous 30 days and pregnant women had been excluded from the research. All patients and control volunteers signed informed written consent forms. The study was conducted in accordance with the Declaration of Helsinki. The protocol was granted authorization by the Institutional Review Board of Medical Research Ethics, Mansoura Faculty of Medicine, Mansoura University (IRB: MS.22.05.2009, date: 31/5/2022). All subjects underwent a complete physical examination and routine biochemical blood lipid profile. Hypertension, and smoking status were reported for patients.

### Biochemical analysis

After an 8-h fast, blood samples were obtained in the morning. A portion of the sample was placed in glass tubes, then allowed to clot at the temperature of the room before being used to calculate serum lipid levels. Enzymatic techniques were used to determine the amounts of TC, TG, HDL-C, and LDL-C in samples using commercially available kits; TC, (BioMed- Cholesterol- LS (#CHO104090), Egypt), TG (Biomed-Triglycerides L.S (#TG117090), (Cairo, Egypt).); HDL-C (BioMed-HDL (#HDL114100).

### Extraction of DNA and genotyping

An additional portion of the sample (3 ml) was added to an EDTA tube and kept at − 80 °C prior to the test, and it was left at room temperature to be employed in DNA extraction. The Easy Pure® Genomic DNA Purification Kit (Cat. No. EE101) was used to recover genomic DNA based on utilizing silica-based membrane technology. The DNA was quantified by ultraviolet light absorption spectrophotometry at 260 nm and a contaminant-free sample was considered when the ratio 260/280 was between 1.7 and 2.0. Each sample was adjusted to 25 ng/µl for subsequent genotyping.

The samples were then analyzed using the Tetra-primer amplification refractory mutation system method, followed by gel electrophoresis, to discover MTHFR polymorphisms^37^. Table 6 lists the primers that had been utilized. MTHFR polymorphisms (rs1801133) and (rs1801131) were tested utilizing an amplification-refractory mutation system-polymerase chain reaction (PCR). Each MTHFR (rs1801133) PCR mixture had a total volume of 40 μl. Each tube contained 8 μl of external primers and 20 µl of master mix ((W10203001), willow fort), which was mixed well with 4 µl of DNA and 8 µl of reverse C and T allele primer. For MTHFR A1298C (rs1801131), two tubes were used for every subject. Each PCR reaction mixture was performed in an overall volume of 40 µl, including 4 µl of common Forword (FC), 4 µl of reverse primer (RC), and 8 µl of reverse A and C allele primer. With 4 µl DNA and 20 µl of master mix in an Eppendorf Gradients Thermal cycle. Samples were amplified using a T-professional thermocycler^37^. The PCR samples were carried out in an Eppendorf Gradient Thermo cycle, then amplified employing a T professional thermocycler (Biometra, Germany). PCR was done with temperature profile as follows: initial denaturation step (95 °C for 5 min), followed by 33 cycles of denaturation (95 °C for 25 s), annealing (60 °C for 30 min) and extension step (72 °C for 25 s), and final extension (72 °C for 10 min) for rs1801133. For rs1801131, initial denaturation step (95 °C for 5 min), followed by 30 cycles of denaturation (95 °C for 30 s), annealing (54.5 °C for 30 min) and extension step (72 °C for 40 s), and final extension (72 °C for 5 min). The PCR products were separated on a 2.5% agarose gel, stained with ethidium bromide, and visualized via UV transillumination (Supplementary Information).

**Table 6:** Primer sequences used for MTHFR (rs1801133) and (rs1801131) polymorphisms.

Mutation	Primer sequence	Size (bp)
MTHFR (rs1801133) c.677C>T	Common F (FO): CCCAGCCACTCACTGTTTTAGTTCAGGC	407
	Common R(RO): GGTGAGAGTGGGGTGGAGGGAGCTTAT	
	R (C allele): CAAAGAAAAGCTGCGTGATGATGAAATAGG	273
	R (T allele): TTGAAGGAGAAGGTGTCTGCGGGCGT	190
MTHFR A1298C (rs1801131)	Common F (FO): 5ʹ-GAAGAAGTTTGCATGCTTGTGGTTG-3ʹ	593
	Common R(RO): 5ʹ-CAGGCAAGTCACCTGGGAGAGA-3ʹ	
	R (A allele): 5ʹ-GGCAAAGAACGAAGACTTCAAAGACACATT-3ʹ	281
	R (C allele): 5ʹ-GAGGAGCTGACCAGTGATGC-3ʹ	361

### Statistical analysis

The statistical package for the social sciences, SPSS version 25 (2017, Armonk, NY: IBM Corp.), was used to perform all statistical analyses. The differences in the distribution of demographic data, clinical characteristics of patients, and MTHFR C677T and A1298C genotypes were determined by the student’s t-test and chi-square test. The estimation of the connection between the MTHFR C677T as well as A1298C profiles and the hazard of stroke was evaluated by Fisher’s exact test, followed by the calculation of an odds ratio (OR) with 95% confidence interval. Statistical tests were considered significant when the P value was less than 0.05^38^. The Haplo-View programmed (version 4.2) was applied to estimate the haplotypes, which uses the expectation maximization (EM) algorithm^39^.

### Ethical approval

This research received ethics approval and consent to participate. The Ethics Board of Mansoura University IRB: MS.22.05.2009, date: 31/5/2022. Samples were obtained with the written informed consent of the corresponding patients.

### Supplementary Information

Supplementary Information.

## Data Availability

The datasets generated and analyzed during the current study are available from the corresponding author on reasonable request.
