# Supplementary figures and images for: Association study between genetic polymorphisms in MTHFR and stroke susceptibility in Egyptian population: a case–control study
Source: Sci Rep. 2024 Jan 2;14:114. doi: 10.1038/s41598-023-50277-z (PMC10762080; doi:10.1038/s41598-023-50277-z)

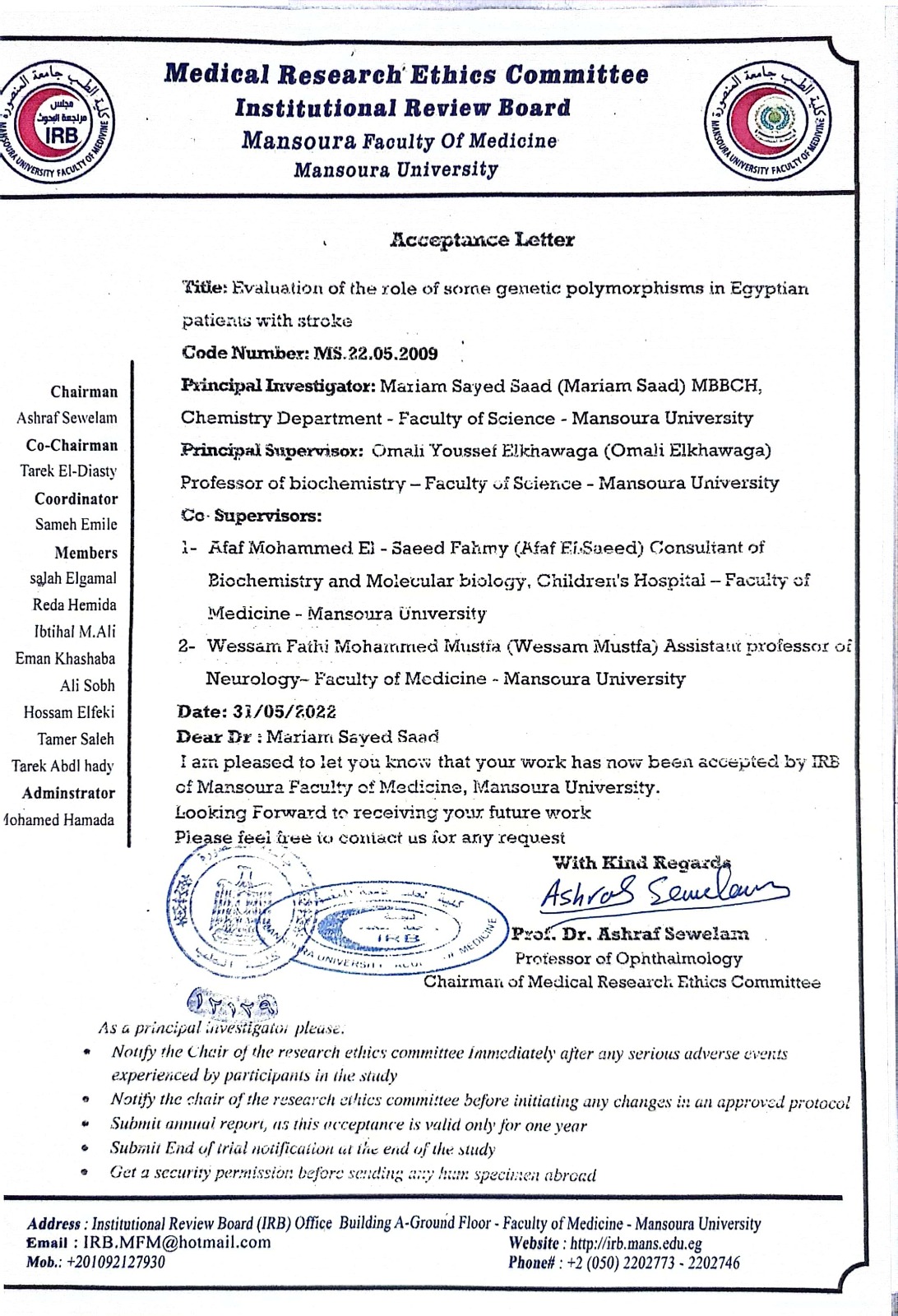

Supplement: Supplementary file 1 — Supplementary Information. [file 41598_2023_50277_MOESM1_ESM.jpg]
